# Diiodido{2-[(4-meth­oxy­phen­yl)imino­meth­yl]pyridine-κ^2^ *N*,*N*′}zinc

**DOI:** 10.1107/S1600536812030486

**Published:** 2012-07-10

**Authors:** Sadegh Salehzadeh, Mehdi Khalaj, Saeed Dehghanpour

**Affiliations:** a Faculty of Chemistry, Bu-Ali Sina University, Hamedan, Iran; b Department of Chemistry, Alzahra University, Tehran, Iran

## Abstract

In the title complex, [ZnI_2_(C_13_H_12_N_2_O)], the Zn^II^ atom has a distorted tetra­hedral coordination. The organic ligand is bidentate, coordinating the Zn^II^ atom *via* the two N atoms. The benzene and pyridine rings are oriented at a dihedral angle of 11.67 (9)°. In the crystal, weak C—H⋯I and C—H⋯O hydrogen bonds are observed, in addition to π–π stacking inter­actions, with a centroid–centroid distance of 3.72 (5) Å.

## Related literature

For the synthesis of the ligand, see: Dehghanpour *et al.* (2009 ▶). For related structures, see: Talei Bavil Olyai *et al.* (2008 ▶); Khalaj *et al.* (2008 ▶); Wriedt *et al.* (2008 ▶).
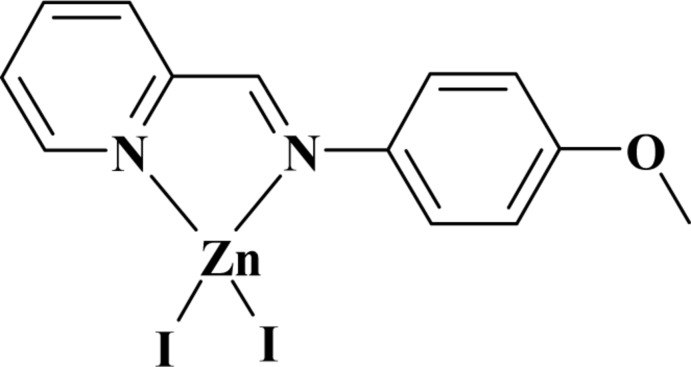


## Experimental

### 

#### Crystal data

[ZnI_2_(C_13_H_12_N_2_O)]
*M* *_r_* = 531.42
Triclinic, 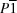

*a* = 8.0290 (15) Å
*b* = 10.002 (2) Å
*c* = 10.538 (2) Å
α = 83.498 (4)°
β = 80.208 (4)°
γ = 71.441 (4)°
*V* = 789.0 (3) Å^3^
*Z* = 2
Mo *K*α radiation
μ = 5.46 mm^−1^
*T* = 150 K
0.25 × 0.12 × 0.08 mm

#### Data collection

Bruker APEX DUO diffractometer
Absorption correction: multi-scan (*SADABS*; Sheldrick, 1996 ▶) *T*_min_ = 0.591, *T*_max_ = 0.746
6595 measured reflections
3584 independent reflections
3165 reflections with *I* > 2σ(*I*)
*R*_int_ = 0.019

#### Refinement

*R*[*F* ^2^ > 2σ(*F* ^2^)] = 0.024
*wR*(*F* ^2^) = 0.061
*S* = 1.03
3584 reflections
173 parameters
H-atom parameters constrained
Δρ_max_ = 0.97 e Å^−3^
Δρ_min_ = −0.96 e Å^−3^

### 

Data collection: *APEX2* (Bruker, 2007 ▶); cell refinement: *SAINT* (Bruker, 2007 ▶); data reduction: *SAINT*; program(s) used to solve structure: *SHELXS97* (Sheldrick, 2008 ▶); program(s) used to refine structure: *SHELXL97* (Sheldrick, 2008 ▶); molecular graphics: *PLATON* (Spek, 2009 ▶); software used to prepare material for publication: *SHELXTL* (Sheldrick, 2008 ▶).

## Supplementary Material

Crystal structure: contains datablock(s) I, global. DOI: 10.1107/S1600536812030486/br2207sup1.cif

Structure factors: contains datablock(s) I. DOI: 10.1107/S1600536812030486/br2207Isup2.hkl

Additional supplementary materials:  crystallographic information; 3D view; checkCIF report

## Figures and Tables

**Table 1:** Hydrogen-bond geometry (Å, °)

*D*—H⋯*A*	*D*—H	H⋯*A*	*D*⋯*A*	*D*—H⋯*A*
C6—H6*A*⋯I2^i^	0.95	3.13	3.761 (3)	125
C1—H1*A*⋯O1^ii^	0.95	2.47	3.338 (4)	152

Symmetry codes: (i) 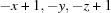; (ii) 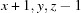.

**Fig. 1.**
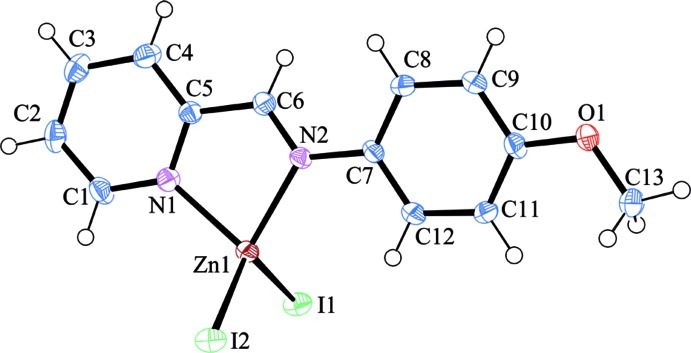
A view of the structure of the title complex, with displacement ellipsoids drawn at the 50% probability level [H atoms are represented as spheres of arbitrary radius].

**Fig. 2.**
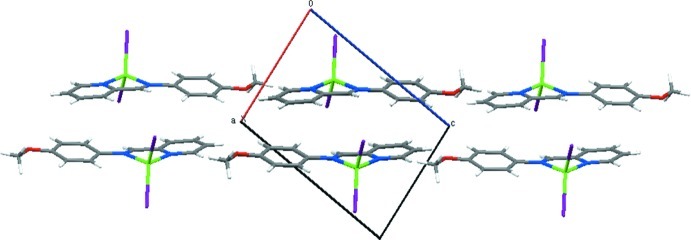
A view of the packing of title molecules along the b axis, in which the Zn, I, N, C and H atoms are shown in green, purple, blue, grey and white balls, respectively.

## supplementary crystallographic information

### Comment

In ongoing our research interest on synthesis and characterization of metal complexes containing bidentate Schiff base ligands (Dehghanpour *et al.* (2009)), here we report structure of the zinc complex of the Schiff base of (4-methoxyphenyl)pyridin-2-ylmethyleneamine. The title complex, (I), was prepared by the reaction of ZnI_2_ with the bidentate ligand (4-methoxyphenyl)pyridin-2-ylmethyleneamine.

The molecular structure of the title complex, and the atom numbering scheme are shown in Fig. 1. The metal centre has a tetrahedral coordination which shows signficant distortion, mainly due to the presence of the five-membered chelate ring. The endocyclic N1—Zn1—N2 angle is much narrower than the ideal tetrahedral angle of 109.5, whereas the opposite I1—Zn1—I2 angle is much wider than the ideal tetrahedral angle. The Zn—I and Zn—N bond dimensions compare well with the values found in other tetrahedral Schiff base adducts of Zinc iodode (Talei Bavil Olyai *et al.* (2008); Khalaj *et al.*, (2008); Wriedt *et al.*, (2008)). In the crystal, weak C—H···I and C—H···O hydrogen bonds are observed in addition to π–π stacking interactions with a centroid to centroid distance of 3.72 (5) Å for *Cg*1···*Cg*2^i^ (where *Cg*1 and *Cg*2 are centroids of the N1—C1—C5 and C7—C12 rings; symmetry code: 1 - *x*, -*y*, 1 - *z*)fig. 2.

### Experimental

The title complex was prepared by the reaction of ZnI_2_ (31.9 mg, 0.1 mmol) and (4-methoxyphenyl)pyridin-2-ylmethyleneamine (21.2 mg, 0.1 mmol) in 15 ml of acetonitrile at room temperature. The solution was allowed to stand at room temperature and yellow crystals of the title compound suitable for X-ray analysis precipitated within few days.

### Refinement

H atoms were placed in calculated positions with C—H = 0.95–0.98 Å and included in the refinement with *U*_iso_(H) = 1.2*U*_eq_(C) or 1.5*U*_eq_(C_methyl_).

### Crystal data

**Table:**

[ZnI_2_(C_13_H_12_N_2_O)]	*Z* = 2
*M**_r_* = 531.42	*F*(000) = 496
Triclinic, *P*1	*D*_x_ = 2.237 Mg m^−^^3^
Hall symbol: -P 1	Mo *K*α radiation, λ = 0.71073 Å
*a* = 8.0290 (15) Å	Cell parameters from 4756 reflections
*b* = 10.002 (2) Å	θ = 2.7–27.5°
*c* = 10.538 (2) Å	µ = 5.46 mm^−^^1^
α = 83.498 (4)°	*T* = 150 K
β = 80.208 (4)°	Needle, yellow
γ = 71.441 (4)°	0.25 × 0.12 × 0.08 mm
*V* = 789.0 (3) Å^3^	

### Data collection

**Table:**

Bruker APEX DUO diffractometer	3584 independent reflections
Radiation source: fine-focus sealed tube	3165 reflections with *I* > 2σ(*I*)
Bruker Triumph monochromator	*R*_int_ = 0.019
φ and ω scans	θ_max_ = 27.6°, θ_min_ = 2.0°
Absorption correction: multi-scan (*SADABS*; Sheldrick, 1996)	*h* = −10→10
*T*_min_ = 0.591, *T*_max_ = 0.746	*k* = −12→12
6595 measured reflections	*l* = −13→13

### Refinement

**Table:**

Refinement on *F*^2^	Primary atom site location: structure-invariant direct methods
Least-squares matrix: full	Secondary atom site location: difference Fourier map
*R*[*F*^2^ > 2σ(*F*^2^)] = 0.024	Hydrogen site location: inferred from neighbouring sites
*wR*(*F*^2^) = 0.061	H-atom parameters constrained
*S* = 1.03	*w* = 1/[σ^2^(*F*_o_^2^) + (0.0321*P*)^2^ + 0.2822*P*] where *P* = (*F*_o_^2^ + 2*F*_c_^2^)/3
3584 reflections	(Δ/σ)_max_ = 0.001
173 parameters	Δρ_max_ = 0.97 e Å^−^^3^
0 restraints	Δρ_min_ = −0.96 e Å^−^^3^

### Special details

**Table:**

Geometry. All e.s.d.'s (except the e.s.d. in the dihedral angle between two l.s. planes) are estimated using the full covariance matrix. The cell e.s.d.'s are taken into account individually in the estimation of e.s.d.'s in distances, angles and torsion angles; correlations between e.s.d.'s in cell parameters are only used when they are defined by crystal symmetry. An approximate (isotropic) treatment of cell e.s.d.'s is used for estimating e.s.d.'s involving l.s. planes.
Refinement. Refinement of *F*^2^ against ALL reflections. The weighted *R*-factor *wR* and goodness of fit *S* are based on *F*^2^, conventional *R*-factors *R* are based on *F*, with *F* set to zero for negative *F*^2^. The threshold expression of *F*^2^ > σ(*F*^2^) is used only for calculating *R*-factors(gt) *etc*. and is not relevant to the choice of reflections for refinement. *R*-factors based on *F*^2^ are statistically about twice as large as those based on *F*, and *R*- factors based on ALL data will be even larger.

### Fractional atomic coordinates and isotropic or equivalent isotropic displacement parameters (Å^2^)

**Table:**

	*x*	*y*	*z*	*U*_iso_*/*U*_eq_	
Zn1	0.29826 (5)	0.21887 (4)	0.31555 (3)	0.02060 (9)	
I1	0.03469 (3)	0.34732 (2)	0.19749 (2)	0.02706 (7)	
I2	0.48121 (3)	0.36778 (2)	0.36178 (2)	0.02624 (7)	
O1	−0.2104 (3)	0.2079 (3)	0.9260 (2)	0.0298 (5)	
N1	0.4611 (3)	0.0328 (3)	0.2380 (2)	0.0209 (5)	
N2	0.2355 (3)	0.0735 (3)	0.4600 (2)	0.0181 (5)	
C1	0.5851 (4)	0.0129 (4)	0.1337 (3)	0.0272 (7)	
H1A	0.6042	0.0931	0.0833	0.033*	
C2	0.6867 (4)	−0.1210 (4)	0.0967 (3)	0.0298 (8)	
H2A	0.7753	−0.1314	0.0231	0.036*	
C3	0.6591 (4)	−0.2385 (4)	0.1666 (3)	0.0296 (7)	
H3A	0.7263	−0.3308	0.1414	0.035*	
C4	0.5294 (4)	−0.2188 (4)	0.2758 (3)	0.0271 (7)	
H4A	0.5066	−0.2975	0.3265	0.033*	
C5	0.4351 (4)	−0.0822 (3)	0.3085 (3)	0.0194 (6)	
C6	0.3046 (4)	−0.0532 (3)	0.4257 (3)	0.0214 (6)	
H6A	0.2717	−0.1283	0.4759	0.026*	
C7	0.1168 (4)	0.1062 (3)	0.5786 (3)	0.0182 (6)	
C8	0.0968 (4)	0.0023 (3)	0.6749 (3)	0.0222 (6)	
H8A	0.1596	−0.0943	0.6620	0.027*	
C9	−0.0142 (4)	0.0400 (3)	0.7885 (3)	0.0244 (7)	
H9A	−0.0283	−0.0309	0.8537	0.029*	
C10	−0.1054 (4)	0.1810 (3)	0.8083 (3)	0.0220 (6)	
C11	−0.0880 (4)	0.2861 (3)	0.7136 (3)	0.0243 (7)	
H11A	−0.1521	0.3825	0.7262	0.029*	
C12	0.0264 (4)	0.2462 (3)	0.5993 (3)	0.0241 (7)	
H12A	0.0422	0.3170	0.5344	0.029*	
C13	−0.2999 (5)	0.3525 (4)	0.9528 (3)	0.0327 (8)	
H13A	−0.3631	0.3573	1.0411	0.049*	
H13B	−0.3849	0.3951	0.8920	0.049*	
H13C	−0.2128	0.4041	0.9436	0.049*	

### Atomic displacement parameters (Å^2^)

**Table:**

	*U*^11^	*U*^22^	*U*^33^	*U*^12^	*U*^13^	*U*^23^
Zn1	0.02370 (17)	0.01803 (19)	0.01862 (18)	−0.00642 (14)	−0.00014 (13)	0.00062 (14)
I1	0.02842 (11)	0.02108 (12)	0.03186 (13)	−0.00595 (8)	−0.00941 (9)	0.00063 (9)
I2	0.03011 (12)	0.02100 (12)	0.02954 (12)	−0.00861 (8)	−0.00965 (8)	0.00107 (9)
O1	0.0389 (13)	0.0246 (13)	0.0197 (12)	−0.0077 (10)	0.0096 (10)	−0.0021 (10)
N1	0.0222 (12)	0.0215 (14)	0.0189 (12)	−0.0063 (10)	−0.0031 (10)	−0.0017 (11)
N2	0.0190 (11)	0.0183 (13)	0.0164 (12)	−0.0058 (10)	−0.0023 (9)	0.0018 (10)
C1	0.0308 (16)	0.0309 (19)	0.0207 (16)	−0.0142 (14)	0.0033 (13)	−0.0015 (14)
C2	0.0252 (15)	0.039 (2)	0.0240 (17)	−0.0089 (14)	0.0051 (13)	−0.0106 (15)
C3	0.0290 (16)	0.0281 (19)	0.0274 (18)	−0.0010 (14)	−0.0028 (13)	−0.0086 (15)
C4	0.0333 (16)	0.0208 (17)	0.0245 (17)	−0.0040 (13)	−0.0052 (13)	−0.0008 (13)
C5	0.0195 (13)	0.0199 (16)	0.0184 (14)	−0.0052 (11)	−0.0046 (11)	0.0013 (12)
C6	0.0237 (14)	0.0206 (16)	0.0183 (15)	−0.0060 (12)	−0.0010 (11)	−0.0004 (12)
C7	0.0177 (13)	0.0200 (15)	0.0165 (14)	−0.0069 (11)	−0.0013 (11)	0.0014 (12)
C8	0.0256 (15)	0.0164 (15)	0.0226 (15)	−0.0049 (12)	−0.0022 (12)	0.0010 (12)
C9	0.0283 (15)	0.0204 (16)	0.0225 (16)	−0.0084 (13)	0.0013 (12)	0.0027 (13)
C10	0.0234 (14)	0.0254 (17)	0.0171 (14)	−0.0092 (12)	0.0003 (11)	−0.0006 (13)
C11	0.0283 (15)	0.0163 (15)	0.0234 (16)	−0.0029 (12)	0.0013 (13)	−0.0006 (13)
C12	0.0293 (15)	0.0200 (16)	0.0209 (15)	−0.0085 (13)	0.0021 (12)	0.0023 (13)
C13	0.0432 (19)	0.0264 (19)	0.0227 (17)	−0.0080 (15)	0.0092 (14)	−0.0058 (14)

### Geometric parameters (Å, º)

**Table:**

Zn1—N1	2.067 (3)	C4—H4A	0.9500
Zn1—N2	2.095 (3)	C5—C6	1.468 (4)
Zn1—I2	2.5326 (5)	C6—H6A	0.9500
Zn1—I1	2.5455 (5)	C7—C12	1.380 (4)
O1—C10	1.377 (3)	C7—C8	1.395 (4)
O1—C13	1.432 (4)	C8—C9	1.376 (4)
N1—C1	1.338 (4)	C8—H8A	0.9500
N1—C5	1.351 (4)	C9—C10	1.388 (4)
N2—C6	1.278 (4)	C9—H9A	0.9500
N2—C7	1.438 (4)	C10—C11	1.389 (4)
C1—C2	1.388 (5)	C11—C12	1.397 (4)
C1—H1A	0.9500	C11—H11A	0.9500
C2—C3	1.373 (5)	C12—H12A	0.9500
C2—H2A	0.9500	C13—H13A	0.9800
C3—C4	1.400 (5)	C13—H13B	0.9800
C3—H3A	0.9500	C13—H13C	0.9800
C4—C5	1.385 (4)		
			
N1—Zn1—N2	80.45 (10)	N2—C6—C5	119.7 (3)
N1—Zn1—I2	110.55 (7)	N2—C6—H6A	120.1
N2—Zn1—I2	118.95 (7)	C5—C6—H6A	120.1
N1—Zn1—I1	114.61 (7)	C12—C7—C8	119.4 (3)
N2—Zn1—I1	111.29 (7)	C12—C7—N2	118.2 (3)
I2—Zn1—I1	116.02 (2)	C8—C7—N2	122.3 (3)
C10—O1—C13	117.6 (3)	C9—C8—C7	119.9 (3)
C1—N1—C5	118.3 (3)	C9—C8—H8A	120.1
C1—N1—Zn1	129.6 (2)	C7—C8—H8A	120.1
C5—N1—Zn1	112.07 (19)	C8—C9—C10	120.4 (3)
C6—N2—C7	121.8 (3)	C8—C9—H9A	119.8
C6—N2—Zn1	111.4 (2)	C10—C9—H9A	119.8
C7—N2—Zn1	126.6 (2)	O1—C10—C9	115.9 (3)
N1—C1—C2	122.1 (3)	O1—C10—C11	123.4 (3)
N1—C1—H1A	118.9	C9—C10—C11	120.7 (3)
C2—C1—H1A	118.9	C10—C11—C12	118.2 (3)
C3—C2—C1	120.0 (3)	C10—C11—H11A	120.9
C3—C2—H2A	120.0	C12—C11—H11A	120.9
C1—C2—H2A	120.0	C7—C12—C11	121.4 (3)
C2—C3—C4	118.2 (3)	C7—C12—H12A	119.3
C2—C3—H3A	120.9	C11—C12—H12A	119.3
C4—C3—H3A	120.9	O1—C13—H13A	109.5
C5—C4—C3	118.7 (3)	O1—C13—H13B	109.5
C5—C4—H4A	120.6	H13A—C13—H13B	109.5
C3—C4—H4A	120.6	O1—C13—H13C	109.5
N1—C5—C4	122.6 (3)	H13A—C13—H13C	109.5
N1—C5—C6	115.5 (3)	H13B—C13—H13C	109.5
C4—C5—C6	121.9 (3)		

### Hydrogen-bond geometry (Å, º)

**Table:**

*D*—H···*A*	*D*—H	H···*A*	*D*···*A*	*D*—H···*A*
C6—H6*A*···I2^i^	0.95	3.13	3.761 (3)	125
C1—H1*A*···O1^ii^	0.95	2.47	3.338 (4)	152

Symmetry codes: (i) −*x*+1, −*y*, −*z*+1; (ii) *x*+1, *y*, *z*−1.
